# Novel markers of MCL1 inhibitor sensitivity in triple-negative breast cancer cells

**DOI:** 10.1016/j.jbc.2024.107375

**Published:** 2024-05-16

**Authors:** Lei Duan, Mehrdad Jafari Tadi, Kelsey M. O'Hara, Carl G. Maki

**Affiliations:** Department of Anatomy and Cell Biology, Rush University Medical Center, Chicago, Illinois, USA

**Keywords:** MCL1 inhibitor, triple negative breast cancer, predictive marker, AXL, ETS1, IL6, EFEMP1

## Abstract

Triple-negative breast cancer (TNBC) is an aggressive breast cancer sub-type with limited treatment options and poor prognosis. Currently, standard treatments for TNBC include surgery, chemotherapy, and anti-PDL1 therapy. These therapies have limited efficacy in advanced stages. Myeloid-cell leukemia 1 (MCL1) is an anti-apoptotic BCL2 family protein. High expression of MCL1 contributes to chemotherapy resistance and is associated with a worse prognosis in TNBC. MCL1 inhibitors are in clinical trials for TNBC, but response rates to these inhibitors can vary and predictive markers are lacking. Currently, we identified a 4-member (*AXL, ETS1, IL6, EFEMP1*) gene signature (GS) that predicts MCL1 inhibitor sensitivity in TNBC cells. Factors encoded by these genes regulate signaling pathways to promote MCL1 inhibitor resistance. Small molecule inhibitors of the GS factors can overcome resistance and sensitize otherwise resistant TNBC cells to MCL1 inhibitor treatment. These findings offer insights into potential therapeutic strategies and tumor stratification for MCL1 inhibitor use in TNBC.

Triple-negative breast cancer (TNBC) is characterized by high heterogeneity, high rates of metastasis, and poor prognosis. Unlike Her2+ and ER+ BC subtypes that can be treated with anti-Her2 or endocrine therapies, TNBC has limited choices. Current treatment options include surgery, chemotherapy, and anti-PDL1 therapy, which are ineffective in advanced cases.

Myeloid-cell leukemia 1 (MCL1) is an anti-apoptotic member of the BCL2 protein family (1). MCL1 is composed of BH1-BH2-BH3 domains that form four hydrophobic binding pockets (P1–P4) responsible for interacting with hydrophobic residues in the BH3 domains of BAK and BIM (1, 2). MCL1 sequesters BAK and BIM to prevent apoptosis. A number of MCL1 inhibitors that block the interaction of MCL1 with BH3 domains have been developed and entered clinical trials (1, 2).

MCL1 is widely expressed in all breast cancers. High expression of MCL1 protein correlates with poor prognosis in TNBC patients (3). MCL1 can promote chemotherapy resistance in TNBC cells, and the MCL1 gene is found co-amplified with MYC in chemoresistant TNBC tumors (4). Targeting MCL1 showed promising results in inhibiting TNBC tumor growth *in vivo*, presenting a potential avenue for therapy in this challenging breast cancer subtype (5). MCL1 inhibitors have entered clinical trials with chemotherapy for TNBC and other advanced solid tumors. However, a challenge for MCL1 inhibitor use is that their effectiveness may vary and there are no molecular markers to identify tumors that will or will not respond.

In the current study, we utilized several public databases to identify potential markers of MCL1 inhibitor sensitivity in TNBC. These included DepMap, the Genomics of Drug Sensitivity in Cancer (GDSC) database, and the cancer cell line encyclopedia (CCLE). By cross-referencing these databases with our own in-house preliminary data, we identified a 4-member gene signature (GS) that can predict MCL1 inhibitor sensitivity in TNBC cell lines. Moreover, knockdown or inhibition of the GS factors enhanced MCL1 inhibitor sensitivity in otherwise resistant cells, while overexpression of the factors promoted resistance in sensitive cells. These findings hold the potential to improve the prediction and effectiveness of MCL1 inhibitors as a treatment for TNBC.

## Results

We wished to identify molecular markers of MCL1 inhibitor sensitivity in TNBC. We hypothesized that by doing so we would gain insight into mechanisms of sensitivity and resistance, and also potential ways to improve MCL1 inhibitor sensitivity. To achieve this, we employed S63845, an MCL1-specific inhibitor currently undergoing clinical trials (also known as S64315/MIK665) with exceptional selectivity (Ki < 1.2 nM, Kd of 0.19 nM) and no noticeable binding to BCL-2 or BCL-xL (Ki> 10,000 nM) (6).

**Figure 1:**
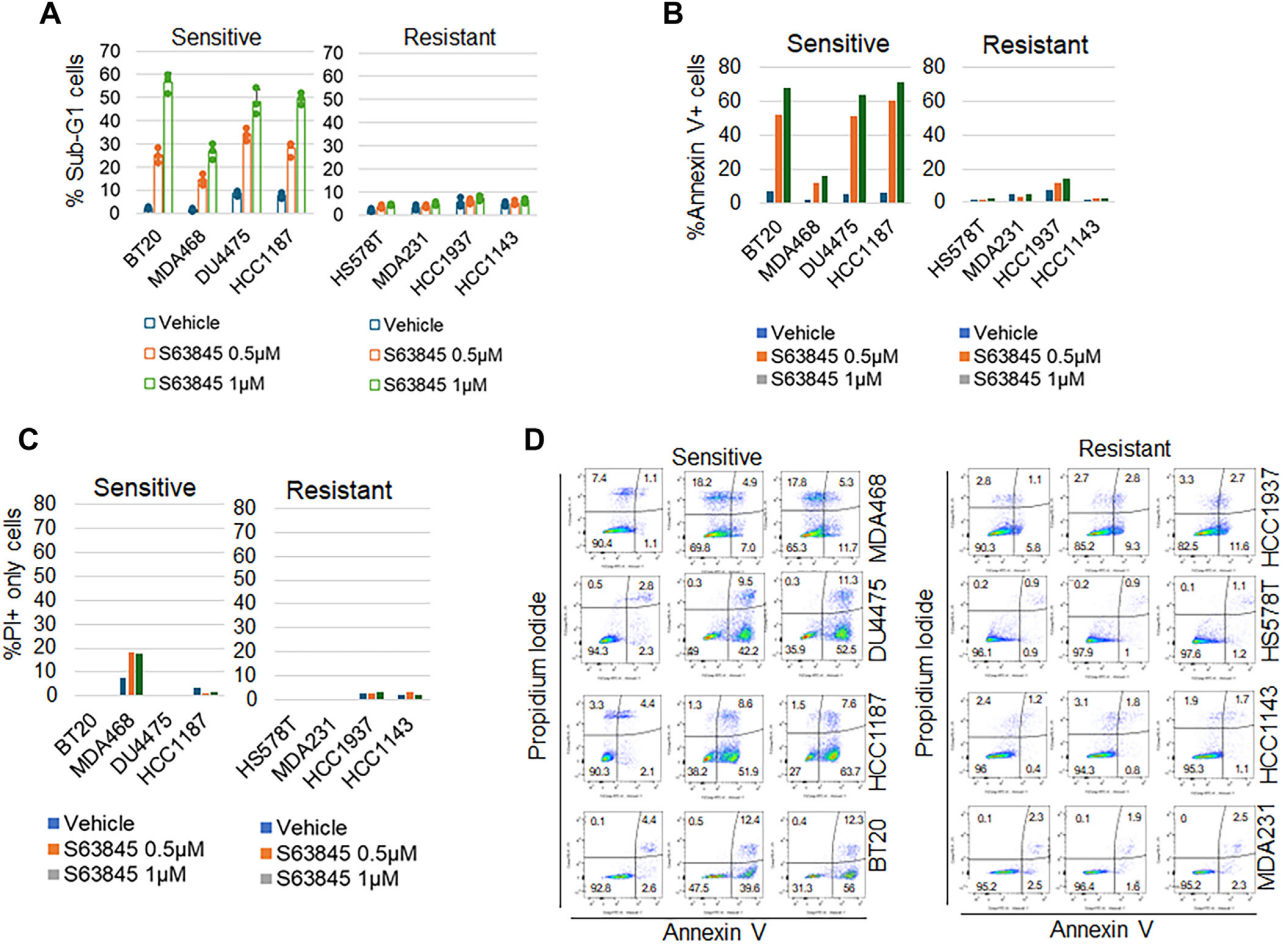
**S63845 sensitive and resistant TNBC cell lines.** The indicated TNBC cell lines were treated with two different doses of S63845 for 3 days. *A*, cell cycle is analyzed for the sub-G1 population. Average (triplicate) % sub-G1 cells were presented as graphs with SD indicated. There are significant differences (*p* < 0.01) between the vehicle and S63845 in sensitive cell lines. No differences in resistant cell lines. Representative results of three independent experiments. *B, C, and D*, cell death is also analyzed with Annexin V and propidium iodide (PI) staining. %Annexin V positive cells (B) and only PI-positive cells (C) are presented (average from two independent experiments).

First, we assessed the response of 8 TNBC cell lines available in our laboratory to S63845. Cells were treated with two different doses of S63845 for 3 days, followed by cell cycle analysis to determine the percentage of sub-G1 cells indicative of apoptosis. Results revealed that four cell lines (BT20, HCC1187, DU4475, and MDA468) exhibited sensitivity to the MCL1 inhibitor, while the remaining four (HCC1937, HCC1143, MDA231, and HS578T) displayed resistance to the treatment (Fig. 1*A*). To examine the effect of S63845 further, we next performed Annexin V and propidium iodide (PI) co-staining to detect apoptotic and necrotic cells, respectively. The analysis demonstrated a large increase in the Annexin V-positive population in BT20, HCC1187, and DU4475 cells, and a less pronounced increase in MDA468 cells (Fig. 1*B*). Notably, S63845 also increased the only PI-positive population (PI+/Annexin V-negative) in MDA468 cells (Fig. 1*C*), suggesting S63845 may kill MDA468 through a combination of apoptosis and necrosis. In contrast, S63845 induced little or no increase in the Annexin V-positive or necrotic (PI+ only) populations in HCC1937, HCC1143, MDA231, and HS578T cells (Fig. 1, *B*–*D*). We used sub-G1 analysis as a marker for cell death in subsequent experiments.

**Figure 2:**
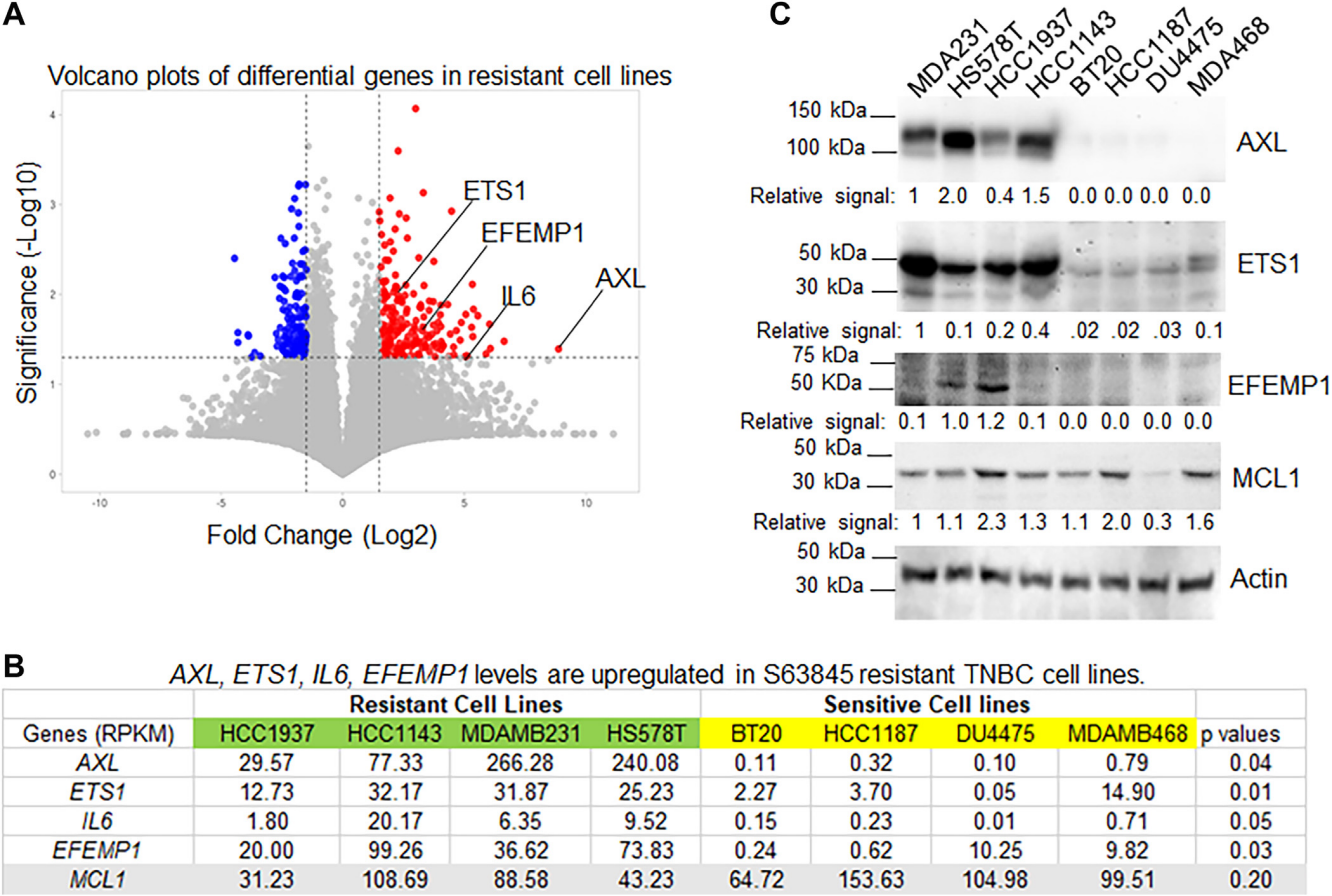
**Identification of commonly upregulated genes in S63845 resistant TNBC cell lines.***A*, volcano plot analysis of genes differentially expressed in resistant vs. sensitive cell lines identified the top 100 commonly upregulated genes in resistant cell lines (gene list seen in Table S1). *B*, 4 genes of interest whose levels (RPKM) are commonly significantly (*p* < 0.05) upregulated in resistant cell lines. Note that MCL1 levels are not different. *C*, lysates of the indicated 8 cell lines were immunoblotted for the indicated proteins with relative signal indicated. Representative results of three independent experiments. Original images are presented in Fig. S2.

To identify potential markers underlying this differential response, we conducted a comparative analysis of gene expression profiles across the different cell lines using RNAseq data from the CCLE. Volcano plot analysis highlighted the top 100 upregulated genes in resistant cell lines (Fig. 2*A* and Table S1). Further examination of these genes revealed four (AXL, IL6, ETS1, and EFEMP1) that were significantly overexpressed in resistant cell lines (depicted in green) compared to sensitive counterparts (depicted in yellow, Fig. 2*B*). These 4 genes are functionally linked as we demonstrate in subsequent experiments. Interestingly, no notable difference in MCL1 expression was observed between the resistant and sensitive groups (Fig. 2, *B* and *C*). Immunoblot analysis corroborated these findings, demonstrating markedly elevated levels of AXL and ETS1 proteins in resistant TNBC cell lines, consistent with the heightened mRNA levels (Fig. 2*C*). EFEMP1 and IL6 were identified as secreted proteins (7, 8). While relatively higher levels of EFEMP1 protein were detected in HS578T and HCC1937 cells compared to others (Fig. 2*C*), IL6 proteins were not detected in cell lysates *via* immunoblotting.

**Figure 3:**
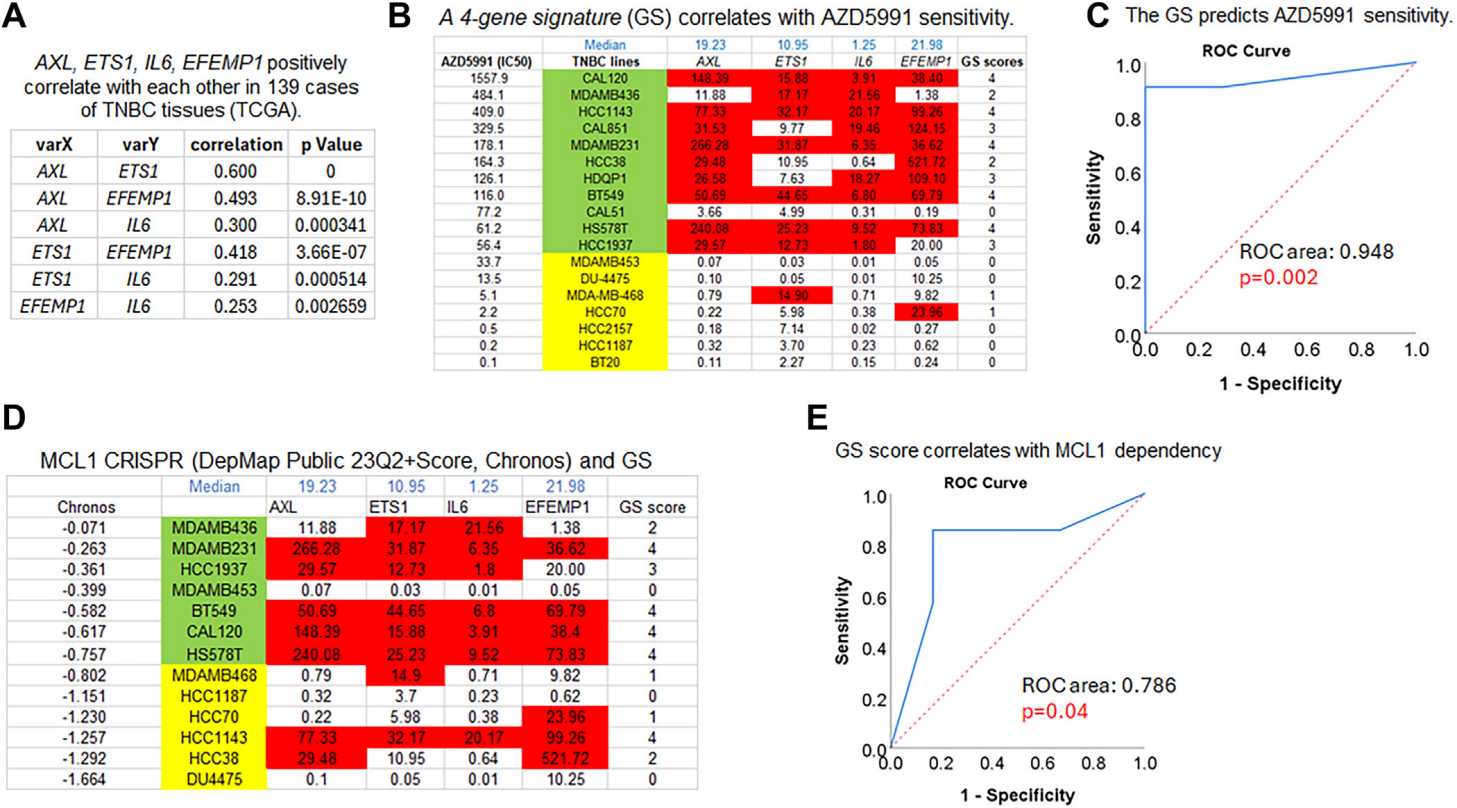
**A 4 gene signature (GS) correlates with AZD5991 sensitivity and MCL-1 dependency in TNBC cell lines.***A*, analysis of gene expression correlation with TIMER software in 139 TNBC tumors (TCGA) showed that the 4 genes positively correlate with each other. *B*, 18 TNBC cell lines from GDSC are divided into AZD5991 resistant (*green*) and sensitive (*yellow*) groups. mRNA levels (RPKM) of AXL, ETS1, IL6, and EFEMP1 in all cell lines were extracted from CCLE RNAseq database. The median level of each gene is calculated and listed on top. Gene levels above the median is marked in *red* and scored 1 and below median is scored 0. The sum of the 4 gene scores is listed on *right*. *C*, correlation of GS scores with AZD5991 sensitivity was analyzed with ROC curve with sensitive cells defined as event 1. The results showed a significant correlation (*p* = 0.002) with an ROC area 0.95 (ROC area 1.0 indicates perfect correlation). D, 13 TNBC cell lines with MCL-1 CRISPR (DepMap public 23Q2) are divided into MCL-1 dependent (*green*) and independent (*yellow*) groups based on their Chromos scores. Gene scores are calculated as described in B. Correlation of GS scores with MCL-1 dependency was analyzed with ROC curve (*E*). The results showed a significant correlation (*p* = 0.04) with a ROC area 0.79).

Next, we explored the expression of these four genes (mRNA) and their relationship in 139 BC tumors using the TCGA dataset and the online TIMER software (Fig. 3*A*). This analysis demonstrated a positive correlation between the expression levels of these genes, indicating their co-expression in different subsets of BC tumors. We hypothesize that the 4 identified genes could serve as a GS for predicting the sensitivity of TNBC to MCL1 inhibitors. To validate this hypothesis, we utilized the GDSC database, which includes 18 TNBC cell lines tested for their response to a different MCL1 inhibitor called AZD5991 that entered clinical trials for hematologic cancers. Based on the IC50 values of AZD5991, we categorized the TNBC cell lines into two groups: relatively sensitive (highlighted in yellow) and resistant (highlighted in green) (Fig. 3*B*). We retrieved the mRNA levels (RPKM) of the four genes from the CCLE database for all the TNBC lines and calculated their median expression levels. Employing a binary system, we assigned a score of 1 for gene expression levels above the median and 0 for levels below the median, resulting in a GS score for each cell line (Fig. 3*B*). Notably, 5 out of 18 TNBC cell lines exhibit higher expression levels for all four genes, whereas 6 out of 18 cell lines show lower expression levels for the same genes. In addition, analysis of the TCGA dataset that includes 152 cases of ER-Her2- BC, we found that 24 out of 152 cases have higher expression of all four genes, and 28 out of 152 cases display lower expression levels for all genes (Table S2), confirming their co-expression in a fraction of TNBC cells/tumors. To assess the predictive capability of the GS, we performed an ROC curve analysis, correlating the GS scores with the sensitivity to AZD5991. The analysis revealed an impressive ROC area of 0.948 (*p* = 0.002) for AZD5991, demonstrating the GS scores' significant ability to predict sensitivity to AZD5991 (Fig. 3*C*).

Next, we used DepMap to further test whether the GS correlates with MCL1 dependence in TNBC cell lines. For this purpose, we downloaded MCL1 CRISPR dependency scores (DepMap Public 23Q2+Score, Chronos) from the Depmap portal, which included a total of 13 TNBC cell lines in the MCL1 CRISPR panel. Based on their dependence scores, we categorized these cell lines into less dependent (green) and more dependent (yellow) groups (Fig. 3*D*). Subsequently, we calculated the GS scores for each cell line as described above and performed ROC curve analysis to assess the correlation between the GS scores and MCL1 dependence. The results demonstrated a significant correlation between the GS scores and MCL1 dependence, with an ROC area of 0.786 (*p* = 0.04) (Fig. 3*E*). Thus, by cross-referencing in-house experiments, the GDSC database, the CCLE RNAseq database, and the DepMap database, we identified a novel GS that predicts MCL1 inhibitor sensitivity and MCL1 dependence in TNBC cell lines.

**Figure 4:**
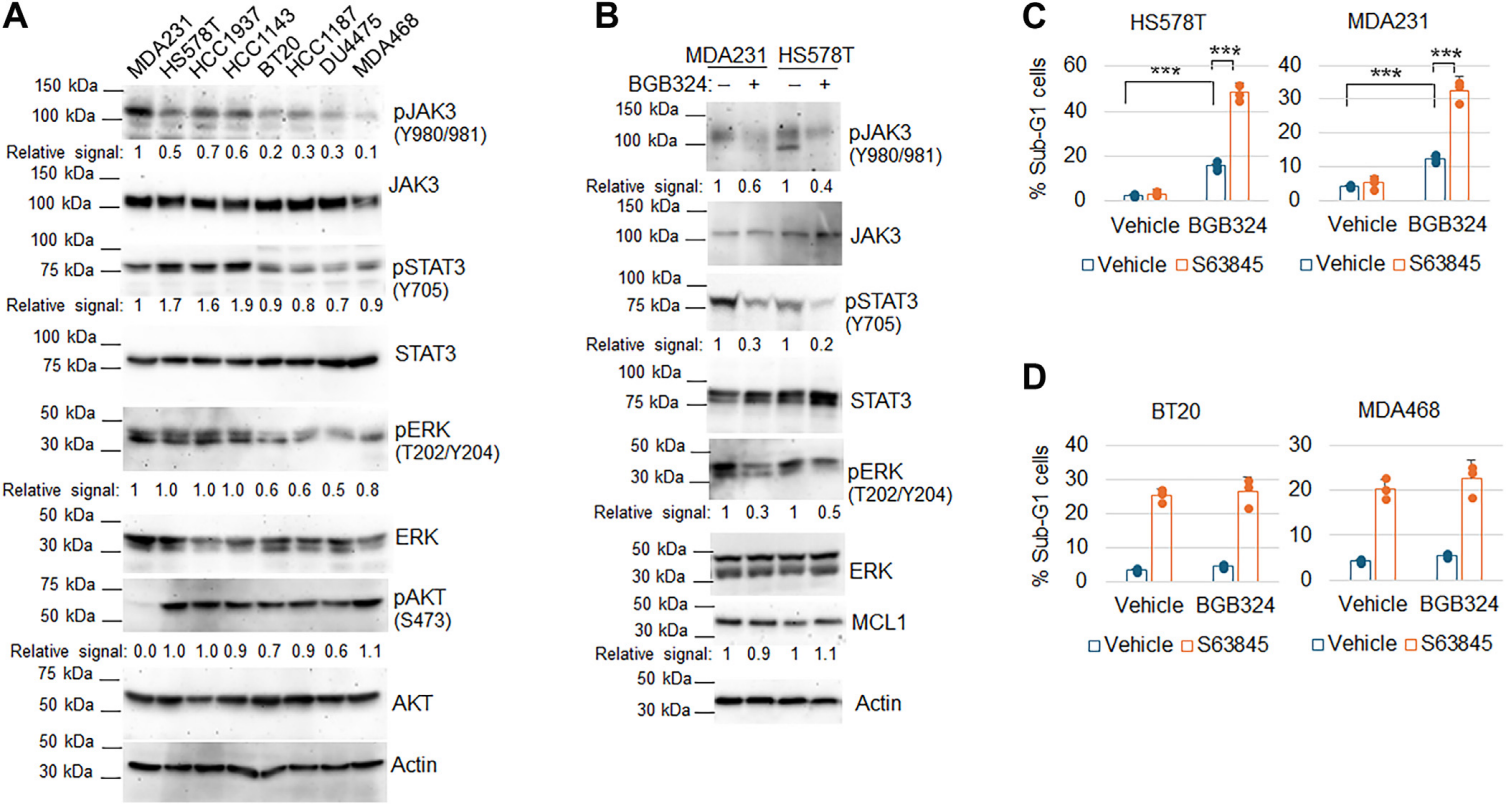
**MCL1 inhibitor-resistant cells have hyper-activation of AXL-JAK/STAT and ERK pathways. AXL inhibitor sensitizes resistant cells.***A*, lysates of the indicated 8 cell lines were immunoblotted for the indicated proteins with relative signals indicated. Representative results of three independent experiments. Original images are presented in Fig. S3. *B*, MDA231 and HS578T cells were treated with vehicle or BG324 (2 μM) for 24 h. Lysates were immunoblotted for the indicated proteins with relative signals indicated. Representative results of three independent experiments. Original images are presented in Fig. S4. *C and D*, the indicated 4 cell lines were treated with vehicle, BGB324 (2 μM), S63845 (1 μM for HS578T, MDA231, and MDA468, 0.5 μM for BT20), or BG324+BGB324 for 3 days and then analyzed for sub-G1 (apoptosis) cells. % sub-G1 cells are presented as graphs. Asterisks indicate statistical significance in sensitive cell lines. No significant differences in resistant cell lines. Representative results of three independent experiments.

We hypothesized high expression of the four GS factors may promote signaling pathways that increase MCL1 inhibitor resistance. AXL is a receptor tyrosine kinase that interacts with Gas6, activating several signaling pathways, including PI3K/AKT/mTOR, JAK/STAT, NF-κB, and RAS/RAF/MEK/ERK (9). IL6 (interleukin 6) is highly expressed in TNBC and promotes JAK/STAT and ERK signaling (10, 11, 12). EGF-containing fibulin-like extracellular matrix protein 1 (EFEMP1) binds to EGFR, triggering EGFR autophosphorylation and activating downstream MAPK and AKT pathways (13). ETS1 is a transcription factor that is activated by ERK and highly expressed in TNBC tumors (14, 15). Immunoblotting showed phospho-JAK3 (Y980/981), phospho-STAT3 (Y705), and phospho-ERK (T202/Y204) proteins, but not phospho-AKT (S473), are higher in resistant versus sensitive cell lines (Fig. 4*A*), suggesting resistant lines have hyper-activated JAK/STAT and RAS/ERK pathways that could contribute to MCL1 inhibitor resistance. Inhibition of AXL with a specific inhibitor BGB324 (currently in clinical trial for AML) downregulated phospho-JAK3, phospho-STAT3, and phospho-ERK in HS578T and MDA231 cells (Fig. 4*B*), indicating AXL activates JAK/STAT and MEK/ERK pathways in these cells. Notably, BGB324 did not affect MCL1 levels (Fig. 4*B*). Moreover, BGB324 significantly increased the percentage of sub-G1 cells in MCL1 inhibitor (S63845)-treated HS578T and MDA231 cells but did not in S63845-treated BT20 and MDA468 cells (Fig. 4, *C* and *D*). These findings support the notion that high AXL expression promotes MCL1 inhibitor resistance in these cells.

**Figure 5:**
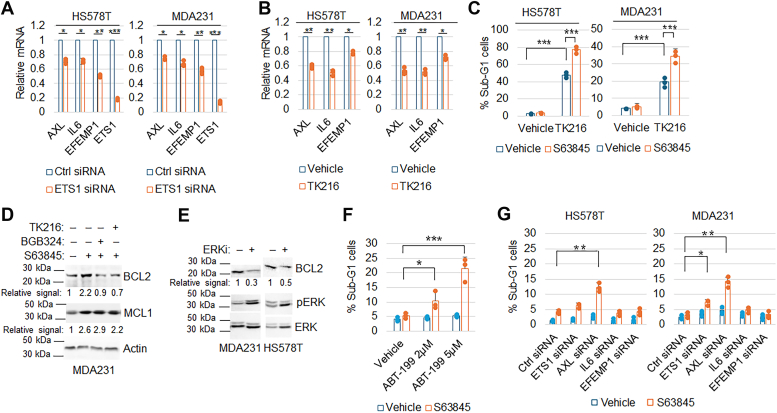
**Inhibition of ETS1 reduces AXL, IL6, and EFEMP1 expression and increases MCL1 inhibitor sensitivity *via* expression of BCL-2.***A*, HS578T and MDA231 cells were transfected with control siRNA or ETS1 siRNA for 24 h. B, HS578T and MDA231 cells were treated with vehicle or TK216 (1 μM) for 24 h. mRNA were qPCR analyzed for the indicated genes. Average (triplicate) relative mRNA is presented with SD indicated. Asterisks indicate statistical significance. Representative results of two independent experiments. *C*, HS578T and MDA231 cells were treated with vehicle, TK216 (1μM), S63845 (1μM), or TK216 + S63845 for 3 days and then analyzed for sub-G1 cells. % sub-G1 cells are presented. Asterisks indicate statistical significance. Representative results of three independent experiments. *D*, MDA231 cells were treated with vehicle, S63845 (1μM), or S63845 plus BGB324 (1 μM) or TK216 (1μM), for 24 h. Lysates were immunoblotted for the indicated proteins. Representative results of two independent experiments. Original images are presented in Fig. S5. *E*, The cells were treated with vehicle or the ERK inhibitor ulixertinib (2 μM) for 24 h. Lysates were immunoblotted for the indicated proteins. Representative results of two independent experiments. Original images are presented in Fig. S6. *F*, MDA231 cells were treated with vehicle, S63845 (1 μM), or S63845 plus ABT-199 (2 μM and 5 μM) for 72 h. *G*, The cells were transfected with control siRNA or the indicated siRNAs against AXL, ETS1, IL6, or EFEMP1 and then treated with vehicle or S63845 for 72 h. Cells were analyzed with FACS for the cell cycle. The average % Sub-G1 cells were presented with SD indicated. Asterisks indicate statistical significance. Representative results of two independent experiments.

ETS1 is a transcription factor and ETS1 gene expression correlates with AXL, IL6, and EFEMP1 in TNBC cell lines and tumor tissues (Fig. 3*A*). This suggested to us that ETS1 may promote their expression. Indeed, data from the Maayanlab website transcription factor database showed that the ETS1 protein binds the promoters of AXL and IL6 (ENCODE Transcription Factor Targets). Knockdown of ETS1 or inhibition of ETS1 with a pan ETS inhibitor TK216 (currently in phase I clinical trial (NCT026570095) for Ewing sarcoma), downregulated AXL, IL6, and EFEMP1 (Fig. 5, *A* and *B*), suggesting ETS1 transcriptionally activates these genes. Moreover, TK216 increased the percentage of sub-G1 (dead) cells in MCL1 inhibitor (S63845)-treated HS578T and MDA231 cells (Fig. 5*C*).

Next, we sought to explore how inhibition of AXL and ETS1 sensitizes resistant cells to S63845. BCL2 is implicated in promoting resistance to MCL1 inhibitors (16), and analysis of the ENCODE database identified BCL2 as a target gene of ETS1. Previous reports suggest that ETS1 enhances BCL2 expression, whereas TK216 suppresses it (17, 18). Given that ERK phosphorylates and activates ETS1, AXL activates ERK downstream (as shown in Fig. 4*B*), and ERK regulates BCL2 expression (19), we hypothesized that AXL and ETS1 may cooperatively regulate BCL2 expression. To investigate this, we treated MDA231 cells with S63845 either alone or in combination with either BGB324 or TK216. Immunoblot analysis revealed an increase in BCL2 expression following treatment with S63845 alone, which was effectively inhibited by co-treatment with BGB324 or TK216 (Fig. 5*D*). Moreover, inhibition of ERK also reduced BCL2 expression (Fig. 5*E*). This observation provides supporting evidence for AXL, ETS1, and ERK signaling in the regulation of BCL2 expression. As anticipated, the specific BCL2 inhibitor ABT-199 sensitized MDA231 cells to S63845-induced apoptosis (Fig. 5*F*), emphasizing the pivotal role of BCL2 in mediating resistance to MCL1 inhibitors facilitated by AXL and ETS1. We next asked if the knockdown of each factor individually could overcome resistance to S63845. Transfection of MDA231 and HS578T cells with control siRNA or siRNAs targeting AXL, ETS1, EFEMP1, or IL6 effectively depleted the respective genes (Fig. S1, *A* and *B*). Subsequent sub-G1 analysis of cells treated with vehicle or S63845 (Fig. 5*G*) demonstrated that AXL depletion significantly increased sensitivity to S63845 in both cell lines. While ETS1 depletion sensitized MDA231 cells, it did not affect HS578T cells' sensitivity to S63845. However, depletion of either EFEMP1 alone or IL6 alone did not impact the cells' response to S63845.

**Figure 6:**
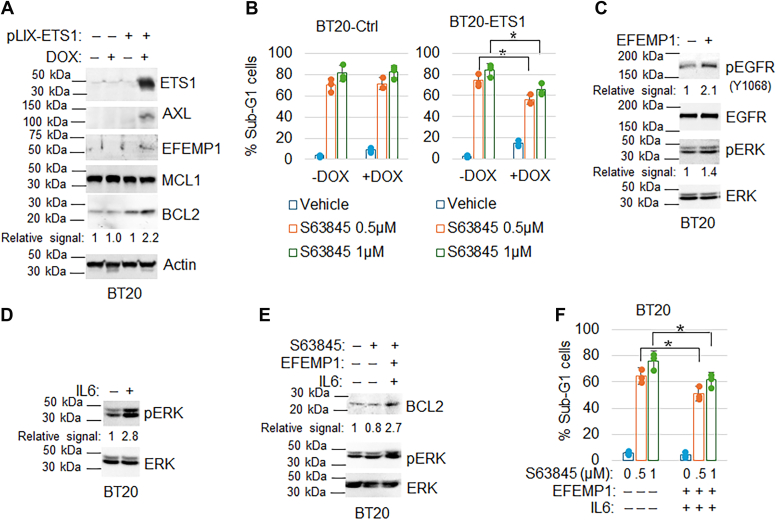
**Overexpression of ETS1 and exogenous EFEMP1 and IL6 increases BCL2 expression and promotes MCL1 inhibitor resistance.***A*, control BT20 cells and BT20-pLIX-ETS1 cells were treated with vehicle or doxycycline (DOX, 0.5 μg/ml) for 24 h. Lysates were immunoblotted for the indicated proteins. Representative images of two independent experiments. *B*, control BT20 cells and BT20-pLIX-ETS1 cells were treated with vehicle or doxycycline (DOX, 0.5 μg/ml) in combination with vehicle or S63845 for 72 h. Cells were analyzed with FACS for cell cycle. Average % Sub-G1 cells (apoptosis) were presented with SD indicated. Asterisks indicate statistical significance. Representative results of two independent experiments. BT20 cells were FBS-starved for 24 h and then treated with vehicle or recombinant EFEMP1 (50 ng/ml) for 30 min (*C*) or recombinant IL6 (50 ng/ml) for 10 min (*D*). *E*, BT20 cells were treated with vehicle or S63845 (1 μM) or S63845 in combination with EFEMP1 plus IL6 for 24 h. Lysates were immunoblotted for the indicated proteins. Representative results of two independent experiments. *F*, BT20 cells were treated with vehicle or S63845 (1 μM) or S63845 in combination with EFEMP1 plus IL6 for 72 h. Cells were analyzed with FACS for the cell cycle. Average % Sub-G1 cells (apoptosis) were presented with SD indicated. Asterisks indicate statistical significance. Representative results of two independent experiments.

To validate the role of the four gene factors in promoting resistance to S63845, we stably transduced the ETS1 gene into BT20 cells, allowing for inducible expression *via* doxycycline. Immunoblot analysis confirmed the induction of ETS1 expression upon doxycycline treatment in ETS1-expressing cells compared to control cells (Fig. 6*A*). Consistent with ETS1 promoting the expression of other genes, we observed increased levels of AXL, EFEMP1, and BCL2 proteins in ETS1-expressing cells upon doxycycline treatment. Notably, MCL1 protein levels remained unchanged in ETS1-expressing cells. Subsequent treatment of control and ETS1-expressing cells with S63845 revealed a significant reduction in sub-G1 cells in response to S63845 in the ETS1-expressing cells compared to controls, confirming that ETS1 promotes resistance to S63845 (Fig. 6*B*).

Next, we examined the effects of EFEMP1 and IL6 on signaling pathways associated with cell survival and resistance. Immunoblot analysis of FBS-starved BT20 cells treated with EFEMP1 or IL6 demonstrated increased phosphorylation of EGFR and a slight increase in ERK phosphorylation in EFEMP1-treated cells, and a more pronounced increase in ERK phosphorylation in IL6-treated cells (Fig. 6, *C* and *D*), consistent with previous reports (13, 20). To examine the role of EFEMP1 and IL6 further, we treated BT20 cells with EFEMP1 or IL6 in the presence of vehicle or S63845. Immunoblot analysis revealed that EFEMP1 and IL6 co-treatment increased BCL2 and phospho-ERK expression in S63845-treated cells (Fig. 6*E*), and reduced the percentage of sub-G1 cells induced by S63845 (Fig. 6*F*). These findings collectively suggest that the four gene factors contribute to S63845 resistance.

**Figure 7:**
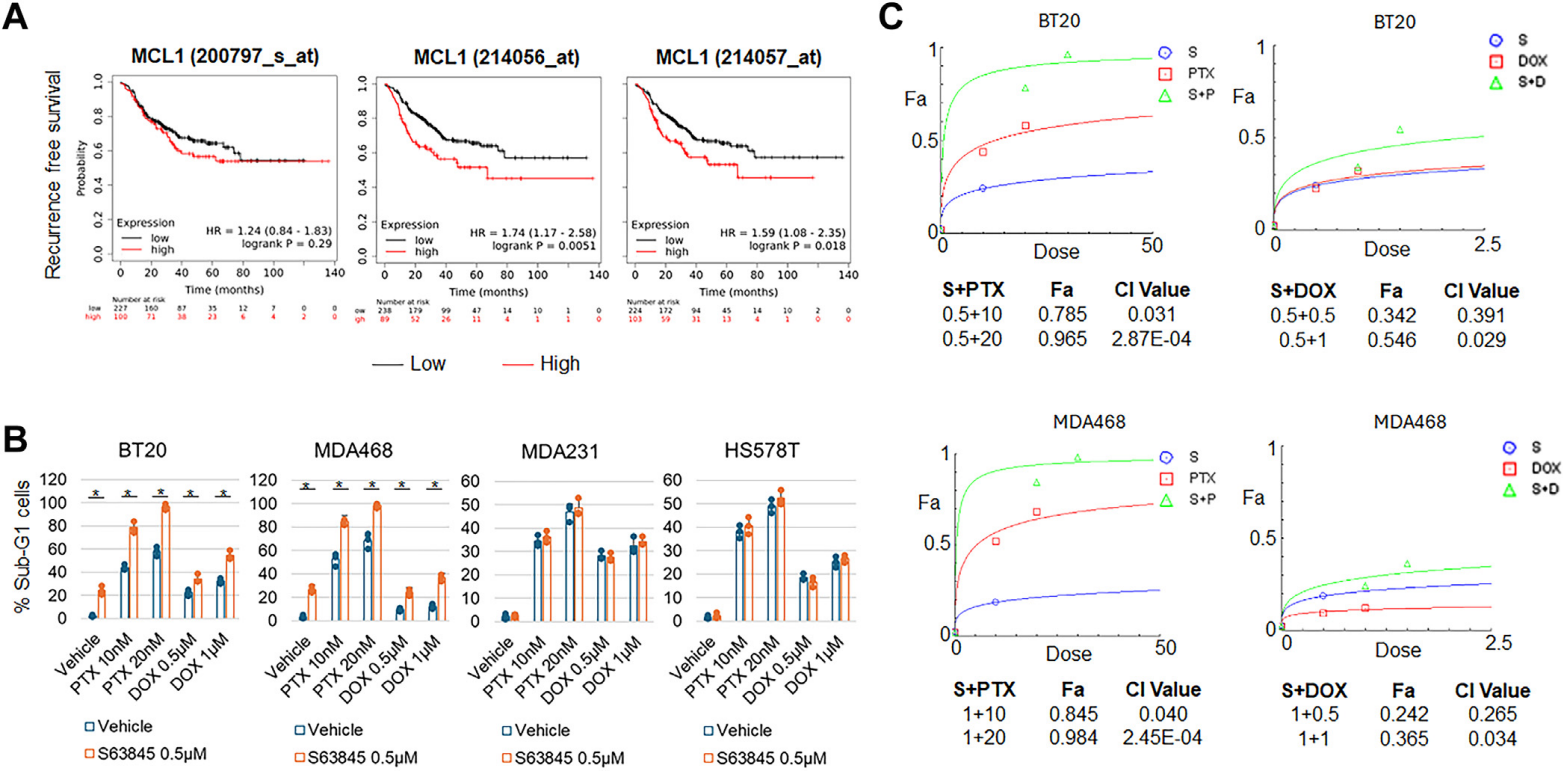
**High expression of MCL1 correlates with chemoresistance and S63845 sensitizes GS low cells to chemotherapy.***A*, MCL1 mRNA (GeneChip) correlation with recurrence-free survival in 327 systemically treated patients with TNBC was analyzed with online Kaplan-Meier Plotter software. Note that two of the three different MCL1 probes show similar results that high MCL1 (automatic cutoff) significantly correlates with reduced RFS, while one probe shows a correlation with reduced RFS that is not significant (*p* < 0.05). *B*, The indicated cell lines were treated with vehicle, paclitaxel (PTX), doxorubicin (DOX), S63845, or a combination of S63845 with PTX or DOX for 3 days. Cells were analyzed for sub-G1. Average (triplicate) % apoptotic cells are presented with SD indicated. There are significant differences (*p* < 0.05) between single drug and combination treatment in BT20 and MDA468 cells but not in MDA231 and HS578T cells. Representative results of three independent experiments. *C*, CompuSyn program was used to analyze the synergistic effects of the combination of S63845 (S, μM) with PTX (nM) or DOX (μM). CI value <1 indicates synergistic effects of the indicated drug combinations.

MCL1 has been associated with chemoresistance in TNBC cell lines (4, 21). To investigate if this observation holds true in clinical patients, we analyzed MCL1 gene expression in 327 TNBC patients treated with systemic chemotherapy using the Kaplan-Meier Plotter database. The results revealed high expression of MCL1 mRNA (GeneChip) in TNBC tissues correlated with reduced recurrence-free survival (RFS) (Fig. 7*A*), providing support for the notion that MCL1 contributes to chemoresistance in TNBC.

Currently, MCL1 inhibitors are being clinically trialed in TNBC in combination with taxanes. We hypothesized that MCL1 inhibition would enhance taxane sensitivity in GS low cells that are responsive to MCL1 inhibitors but may not sensitize GS high cells that are resistant to MCL1 inhibitors. To test this, we treated GS low cells (BT20 and MDA468) and GS high cells (HS578T and MDA231) with either vehicle, S63845 (MCL1 inhibitor), paclitaxel (PTX), doxorubicin (DOX), or a combination of S63845 and either PTX or DOX. The percentage of cells with sub-G1 DNA content was used as an indicator of cell death. The results demonstrated that S63845 sensitized GS low cells (BT20 and MDA468) to PTX and DOX treatment but did not sensitize GS high (MDA231 and HS578T) cells (Fig. 7*B*). CompuSyn analysis showed that the combination of S63845 with PTX or DOX has a synergistic effect (Fig. 7*C*). The results suggest the GS could be used as a predictive marker to determine the response of TNBC cells to the combination of MCL1 inhibitors with chemotherapy. It is important to note that higher expression levels of AXL and ETS1 do not show a correlation with patient outcomes (Fig. S2). Conversely, an increase in IL6 expression is associated with poorer outcomes, whereas elevated levels of EFEMP1 are linked to improved outcomes (Fig. S2). These findings imply that while the four GS may be predictive of sensitivity to MCL1 inhibitors, it does not predict the efficacy of chemotherapy. This assertion is supported by the observation that cells resistant to MCL1 inhibitors exhibit comparable sensitivity to doxorubicin treatment, as shown in Fig. 7*B*.

## Discussion

MCL1 plays a critical role in TNBC cell survival and therapy resistance, making it an attractive target for therapeutic intervention. Several MCL1 inhibitors have been developed and are currently being investigated in clinical trials for TNBC and other solid tumor types. However, TNBC is known for its high heterogeneity, and the precise molecular mechanisms underlying its response to MCL1 inhibitors remain unclear. In our current study, we aimed to elucidate novel molecular mechanisms that govern TNBC's response to MCL1 inhibitors and identify potential markers that can predict sensitivity to these inhibitors. By doing so, we hope to enhance our understanding of TNBC's complex response to MCL1-targeted therapy and ultimately improve treatment outcomes for patients with this aggressive subtype of breast cancer.

In our studies, we initially identified four TNBC cell lines (BT20, HCC1187, DU4475, and MDA468) that exhibited sensitivity to the MCL1 inhibitor S63845, while four other cell lines (HCC1937, HCC1143, MDA231, and HS578T) demonstrated resistance to the inhibitor. Through comprehensive gene expression analysis of these cell lines, we discovered that four genes (ETS1, AXL, IL6, and EFEMP1) exhibited significantly higher expression in the resistant cell lines compared to the sensitive ones. Immunoblot analysis confirmed that AXL and ETS1 proteins are highly expressed in the 4 resistant cell lines compared to the four sensitive cell lines. Furthermore, we conducted a thorough analysis of gene expression correlation in clinical TNBC tumors, which revealed a positive correlation between the expression levels of the four genes, providing evidence that these genes collectively can form a GS that is potentially relevant in identifying subsets of TNBC cells and tumors. These findings led us to hypothesize that this GS might be indicative of MCL1 inhibitor sensitivity in subsets of TNBC cells.

To validate the predictive value of the GS, we turned to the GDSC database, which contains information on TNBC cell line responses to another MCL1 inhibitor, namely AZD5991 that also entered clinical trials. Utilizing the IC50 values as a measure of sensitivity, we divided the TNBC cell lines into two groups: resistant and sensitive. Then, we obtained the gene expression levels of the four genes from the CCLE database and calculated the median levels for each gene, subsequently scoring the GS in each TNBC cell line. By examining the correlation between the GS scores and sensitivity using ROC curve analysis, we observed that high GS scores were significantly associated with resistance of the cell lines to AZD5991. Furthermore, by analyzing MCL1 dependence in the Depmap database, we found that the GS also significantly correlates with MCL1 dependence in TNBC cell lines. These results strongly suggest that the 4-member GS holds the potential as a reliable predictor of MCL1 inhibitor sensitivity in TNBC cell lines. It is important to note that the 4-member GS we identified does not align with any specific TNBC subtype classified by Lehmann BD, *et al.* (22). Among the 8 TNBC cell lines tested, HCC1937, HCC114, and MDA468 belong to the basal-like 1 subtype, MDA231 and HS578T belong to the Mesenchymal-like subtype, DU4475 and HCC1187 belong to the Immunomodulatory subtype, while BT20 falls under the unclassified subtype. This indicates that the GS is not restricted to any particular molecular subtype of TNBC and may have broader implications in predicting sensitivity to MCL1 inhibitors across diverse TNBC subtypes.

These four genes, namely, AXL, IL6, EFEMP1, and ETS1, may functionally contribute to MCL1 resistance by regulating key signaling pathways. AXL, a receptor tyrosine kinase, has been shown to activate multiple pathways, including PI3K/AKT/mTOR, JAK/STAT, NF-κB, and RAS/RAF/MEK/ERK (9). IL6, an interleukin, activates IL6R to promote JAK/STAT and ERK signaling (10, 11, 12). EFEMP1 binds to EGFR, promoting EGFR-mediated MAPK and AKT pathways (13). Consistent with this, the four resistant cell lines exhibit higher JAK/STAT and ERK activation, as indicated by elevated levels of phosphorylated/activated JAK3, STAT3, and ERK in these cells. Furthermore, ETS1 is a transcriptional factor activated downstream of ERK and may impact signaling gene networks, influencing the response to MCL1 inhibitors. Analysis of public databases revealed a positive correlation between ETS1 and the expression levels of AXL, IL6, and EFEMP1 in clinical TNBC tumors. Additionally, data from the ENCODE database indicates that ETS1 binds the promoters of AX and IL6. Inhibition or knockdown of ETS1 decreased the expression of AXL, IL6, and EFEMP1, while overexpression of ETS1 increased their expression. Moreover, inhibition of AXL, ETS1, and ERK downregulated BCL2 and sensitized otherwise resistant cells to S63845, while overexpression of ETS1 or application of exogenous EFEMP1 and IL6 increased BCL2 expression and reduced S63845-induced cell death in sensitive BT20 cells, suggesting that high expression of these four genes not only serves as predictive markers of MCL1 resistance but also form a signaling network that functionally promotes resistance, at least in one way *via* promoting BCL2 expression, and can be targeted to enhance the sensitivity of MCL1 inhibitors. Additionally, the combination of MCL1 inhibitors with taxanes has the potential to increase sensitivity in GS low cells, while not sensitizing GS high cells to MCL1 inhibitors in clinical trials.

In conclusion, our study provides valuable insights into the predictive value of a 4-member gene signature (AXL, IL6, ETS1, and EFEMP1) for determining sensitivity to MCL1 inhibitors, both individually and in combination with taxanes, in TNBC cell lines. Additionally, we have identified these genes as key regulators of signaling pathways associated with MCL1 inhibitor resistance, making them potential therapeutic targets to sensitize resistant cells. By gaining a better understanding of the molecular mechanisms underlying TNBC's response to MCL1 inhibitors, these findings have the potential to enhance the efficacy of targeted therapy for this aggressive subtype of breast cancer.

## Experimental procedures

### Cells and reagents

BT20, HCC1187, DU4475, MDA468, HCC1937, HCC1143, MDA231, and HS578T breast cancer cell lines were obtained from ATCC. All cell lines were grown in RPMI medium, with 10% fetal bovine serum (FBS), penicillin (100 U/ml), and streptomycin (100 μg/ml). Cells were plated 24 h before treatment with different drugs at the indicated concentrations. S63845, BGB324, TK216, and paclitaxel were obtained from Selleck Chemicals. Recombinant EFEMP1 and IL6 proteins are obtained from R&D systems.

### Gene expression omnibus and CCLE RNAseq databases and bioinformatics analysis

GEO datasets contain 491 TNBC cases treated with systemic chemotherapy with recurrence-free survival data available (23, 24). The dataset was analyzed using the Kaplan-Meier Plotter software and database (http://kmplot.com/analysis/) as previously described. The auto-select best cutoff option was used to divide patients into high vs. low expression of MCL1. Kaplan-Meier survival curves were plotted to compare recurrence-free survival times between high vs. low expression of MCL1. Log-rank test was used to determine the significance between groups. Analysis of the CCLE RNAseq database is previously described (25). Briefly, the CCLE RNAseq gene expression (RPKM) database (26) for 1019 cell lines (CCLE RNAseq genes rpkm 20180929.gct.gz) was downloaded from the Broad Institute CCLE database. Differential gene expression in resistant vs. sensitive cell lines were analyzed with the online VolcaNoseR-Exploring volcano plots program according to the instructions. The criteria for differences are set with log2_FoldChange at 1.5 and minus_log10_pvalue at 1.3.

ETS1 target gene list is accessible at the link: https://maayanlab.cloud/Harmonizome/gene_set/ETS1/ENCODE+Transcription+Factor+Targets.

### Analysis of gene correlation using the Tumor IMmune Estimation Resource (TIMER)

The TIMER website (https://cistrome.shinyapps.io/timer/) was used to analyze gene correlation in invasive breast cancer patients from the TCGA database (27). It contains 139 cases of TNBC. Images and statistical analyses are automatically generated by the onsite software.

### Immunoblotting

Whole-cell extracts were prepared by scraping cells in Triton x-100 lysis buffer, resolved by SDS-PAGE and transferred to polyvinylidene difluoride membranes (Thermo Fisher Scientific). Antibodies to BCL2 (124), MCL1 (D35A5), pAKT (S473) (D9E), AKT (11E1), pJAK3 (Y980/981) (D44E3), JAK3 (D7B12), pSTAT3 (Y705) (D3A7), STAT3, pERK1/2 (T202/Y204) (197G2), ERK1/2 (137F5), AXL (C89E7) and ETS1 (D808A) were from Cell Signaling; EFEMP1 (fibulin-3, mAb35) and β-actin (C4) antibodies were from Santa Cruz. Primary antibodies were detected with goat anti-mouse or goat anti-rabbit secondary antibodies conjugated to horseradish peroxidase from Invitrogen using *Immobilon Western HRP Substrate* from EMD Millipore (Burlington, MA). Experiments were conducted three times with representative one presented.

### Flow cytometry

For cell cycle analysis, cells were harvested and fixed in 25% ethanol overnight. The cells were then stained with propidium iodide (25 μg/ml, Calbiochem). Flow cytometry analysis was performed on a Gallios Flow Cytometer (Beckman Coulter), analyzed with FlowJo 10 (Treestar Inc). For each sample, 10,000 events were collected. Experiments are conducted in triplicate and repeated at least one more time. The average value from one representative experiment is presented with SD indicated as error bars.

For FACS analysis of Annexin V and propidium stained cells, cells were trypsinized and washed with 1× PBS before staining using Annexin V-FITC Apoptosis Detection Kit (Cell Signaling, #6592) according to manufacturer's protocol. Cells were filtered through a 35 uM cell strainer and promptly ran on a BD FACSCanto II. Gates were set for live cells and singlets, and results were analyzed on FlowJo 10.10.0.

### Lentiviral transduction

The pLIX-ETS1 is a generous gift from Dr Basabi *Rana*. The lentiviral packaging and envelop vectors psPAX2 and pMD2.G (Addgene plasmid 12260 and 12259 deposited by Dr Didier Trono) were obtained from the Addgene plasmid repository. Lentiviral supernatants for the expression of ETS1 were generated from 293FT cells by co-transfection of shRNA constructs with psPAX2 and pMD2.G packaging and envelope vectors according to the OpenBiosystems protocol. BT20 cells were infected and selected with puromycin (2 μg/ml) for 2 weeks to establish polyclonal lines.

### siRNA-mediated transient knockdown

ETS1, AXL, IL6, EFEMP1 siRNAs (On-target plus smart pool) and Control siRNA (On-target plus siControl non-targeting pool) were purchased from GE Dharmacon and were transfected according to the manufacturer's guidelines using DharmaFECT I reagent.

### RNA isolation and Real-time quantitative PCR analysis

Total RNA was prepared using Total RNA Mini Kit (IBI Scientific); the first cDNA strand was synthesized using High Capacity cDNA Reverse Transcription Kit (Applied Biosystems). Manufacturers’ protocols were followed in each case. PCR primers are listed in Table S3. *SYBR* green PCR kit (*Midwest Scientific*) was used according to the manufacturer’s instructions. QuantStudio 6 was used as follows: activation at 95 °C; 2 min, 40 cycles of denaturation at 95 °C; 15 s and annealing/extension at 60 °C; 60 s, followed by melt analysis ramping from 60 °C to 95 °C. Relative gene expression was determined by the ΔΔC_t_ method using β-Actin to normalize. Experiments are conducted in triplicate and repeated at least one more time. The average value from one representative experiment is presented with SD indicated as error bars.

### Statistical analysis

ROC curve analysis was conducted with IBM SPSS software. One-way analysis of variance (ANOVA) and Student's *t* test were used to determine the statistical significance of differences among experimental groups. Student's *t* test was used to determine the statistical significance between the control and experimental groups.

## Data availability

All the CCLE-processed datasets are available at the CCLE portal (www.broadinstitute.org/ccle). MCL1 gene dependency data are available through the DepMap portal (http"//www.depmap.org) and cell line sensitivity IC50 data to the MCL1 inhibitor AZD5991 is available at the Genomics of Drug Sensitivity in Cancer site (https://www.cancerrxgene.org/). TCGA data in this study is available in Table S2. All other data are held by the corresponding author's institution and will be made available upon reasonable request.

## Supporting information

This article contains supporting information.

## Conflict of interest

The authors declare that they have no conflicts of interest with the contents of this article.
