# Supplementary material for: Novel markers of MCL1 inhibitor sensitivity in triple-negative breast cancer cells
Source: J Biol Chem. 2024 May 16;300(6):107375. doi: 10.1016/j.jbc.2024.107375 (PMC11208921; doi:10.1016/j.jbc.2024.107375)

Supplemental data

Supplemental figure legend

Figure S1. Analysis of siRNA knockdown of the four genes. HS578T and MDA231 cells were transfected with control siRNA or siRNAs for AXL, ETS1, IL6, and EFEMP1 respectively. After 24 hrs, cells were harvested. Proteins were immunoblotted for the indicated proteins (A and B). mRNA was analyzed with for the indicated genes (C and D).

Figure S2. **Correlation of AXL, ETS1, IL6, and EFEMP1 with outcomes in chemotherapy treated TNBC patients.** AXL. ETS1, IL6, and EFEMP1 mRNA (GeneChip) correlation with recurrence free survival in 327 systemically treated TNBC patients was analyzed with online Kaplan-Meier Plotter software.

Figure S3. Original whole images for Figure 2C are presented.

Figure S4. Original whole images for Figure 4A are presented.

Figure S5. Original whole images for Figure 4B are presented.

Figure S6. Original whole images for Figure 5D are presented.

Figure S7. Original whole images for Figure 6C are presented.

Figure S8. Original whole images for Figure 6A are presented.

Figure S9. Original whole images for Figure 6D and E are presented.

Figure S10. Original whole images for Figure 6F are presented.

Figure S1

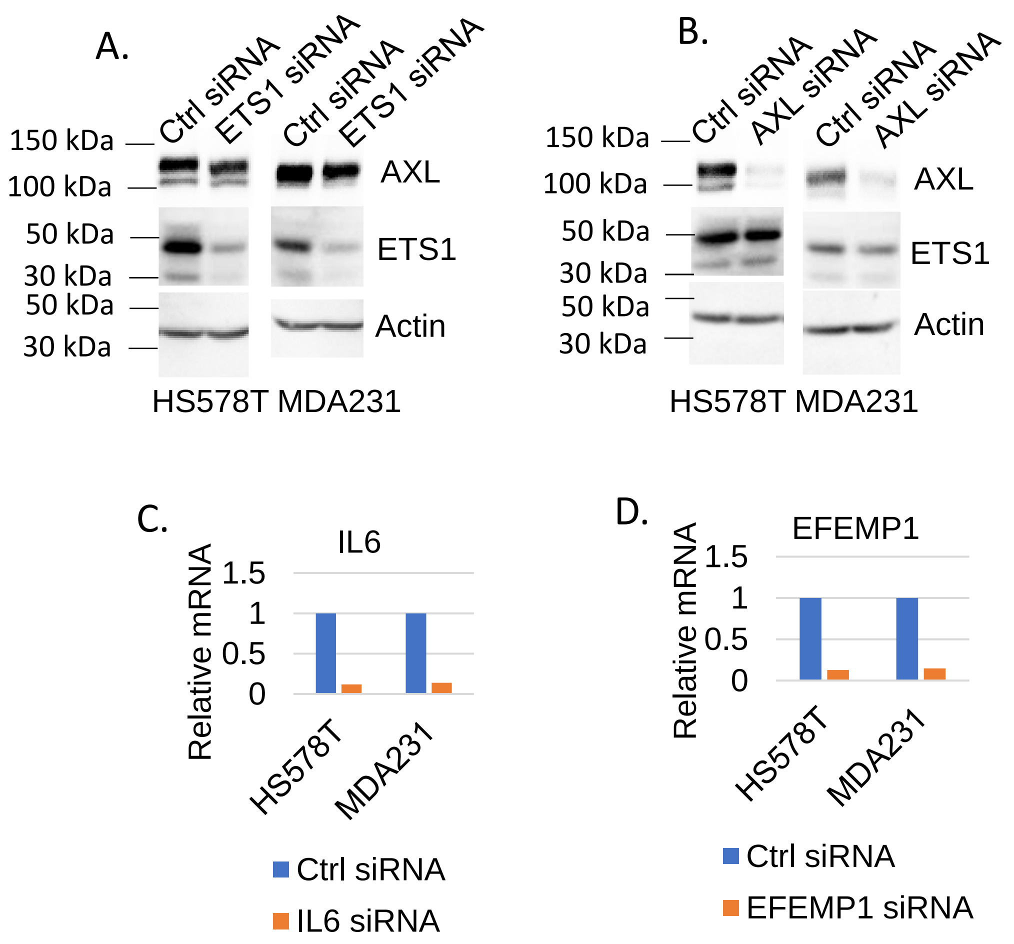
A.
Ctrl siRNA
ETS1 siRNA
Ctrl siRNA
ETS1 siRNA
150 kDa
100 kDa
50 kDa
30 kDa
50 kDa
30 kDa
AXL
ETS1
Actin
HS578T MDA231
B.
Ctrl siRNA
AXL siRNA
Ctrl siRNA
AXL siRNA
150 kDa
100 kDa
50 kDa
30 kDa
50 kDa
30 kDa
AXL
ETS1
Actin
HS578T MDA231
C.
IL6
Relative mRNA
1.5
1
0.5
0
HS578T
MDA231
Ctrl siRNA
IL6 siRNA
D.
EFEMP1
Relative mRNA
1.5
1
0.5
0
HS578T
MDA231
Ctrl siRNA
EFEMP1 siRNA

Figure S2

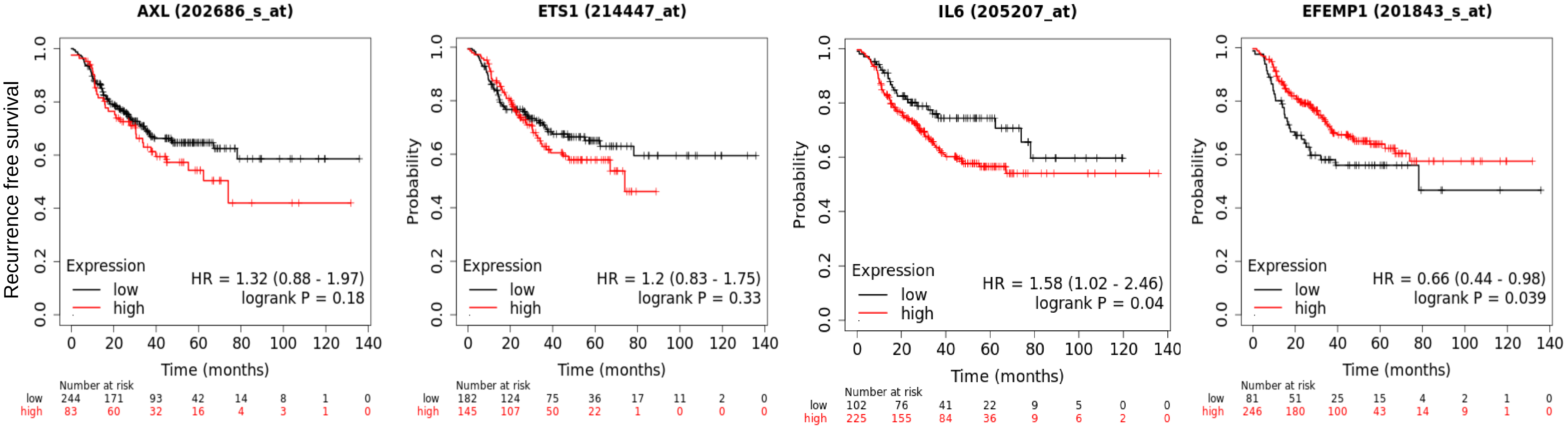

AXL (202686_s_at)
Recurrence free survival
Expression
low
high
HR = 1.32 (0.88 - 1.97)
logrank P = 0.18
Time (months)
Number at risk
low 244 171 93 42 14 8 1 0
high 83 60 32 16 4 3 1 0
ETS1 (214447_at)
Probability
Expression
low
high
HR = 1.2 (0.83 - 1.75)
logrank P = 0.33
Time (months)
Number at risk
low 182 124 75 36 17 11 2 0
high 145 107 50 22 1 0 0 0
IL6 (205207_at)
Probability
Expression
low
high
HR = 1.58 (1.02 - 2.46)
logrank P = 0.04
Time (months)
Number at risk
low 102 76 41 22 9 5 0 0
high 225 155 84 36 9 6 2 0
EFEMP1 (201843_s_at)
Probability
Expression
low
high
HR = 0.66 (0.44 - 0.98)
logrank P = 0.039
Time (months)
Number at risk
low 81 51 25 15 4 2 1 0
high 246 180 100 43 14 9 1 0

Figure S3

Whole images of immunoblots for Fig 2C

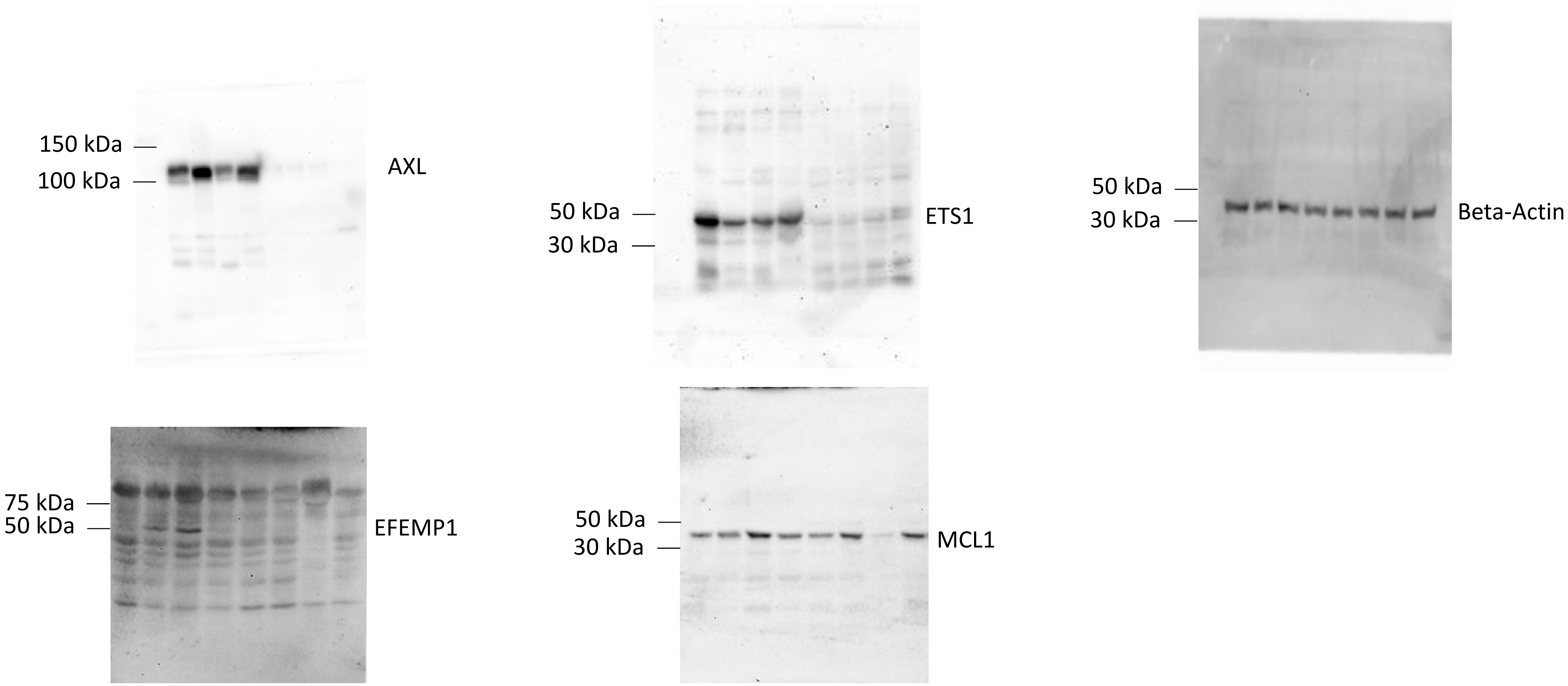

Figure S4

Whole images of immunoblots for Fig 4A

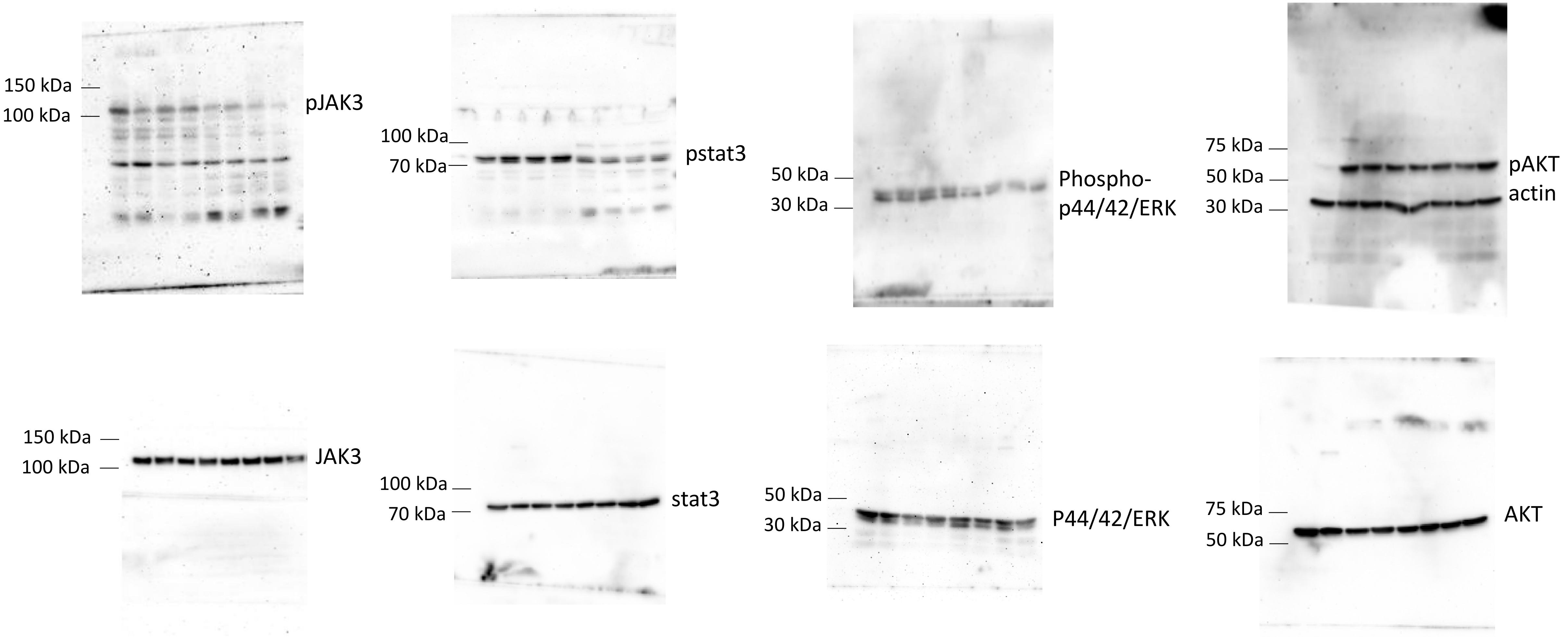

Figure S5

Whole images of immunoblots for Fig 4B

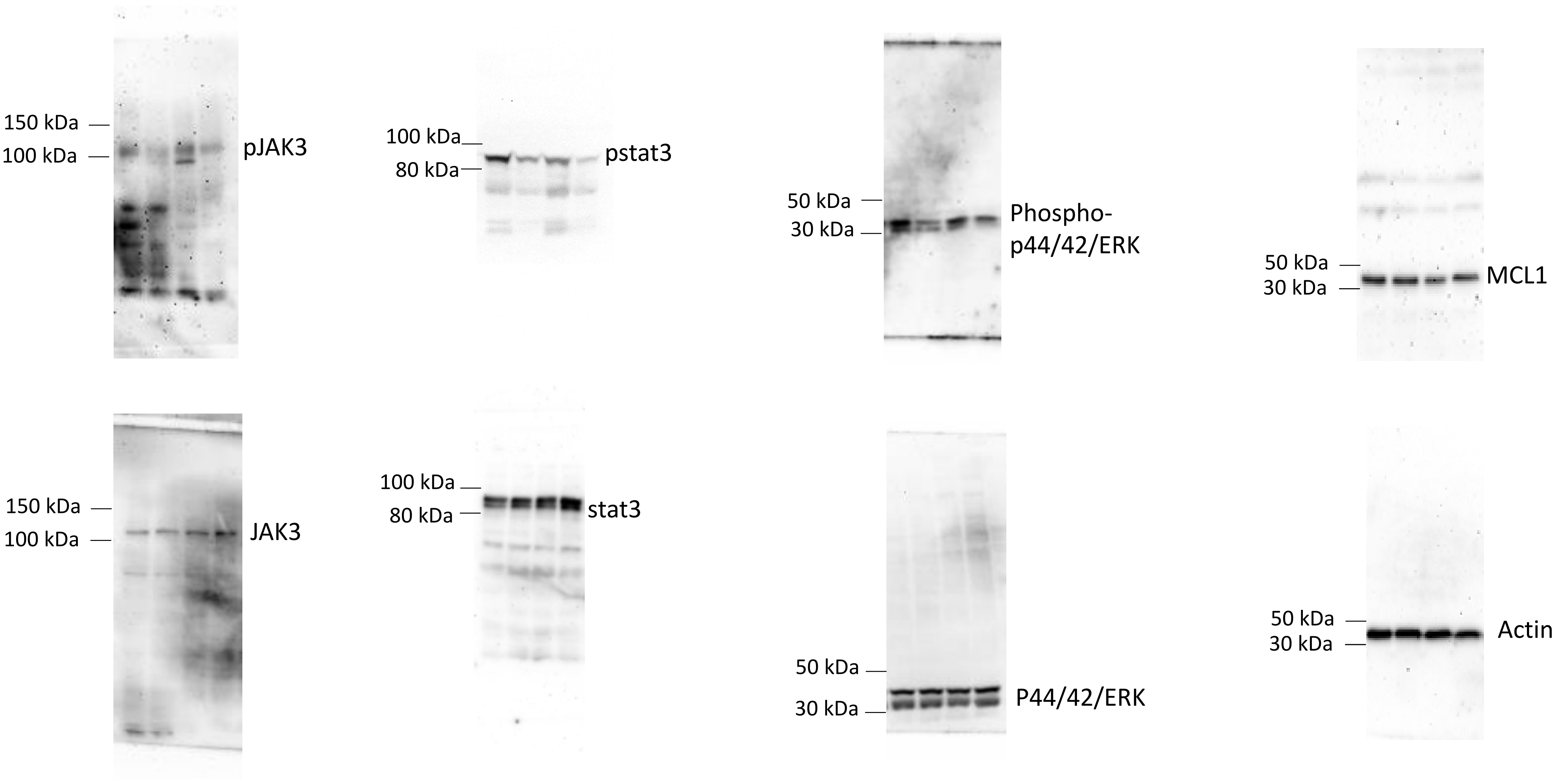

Figure S6

Whole images of immunoblots for Fig 5D

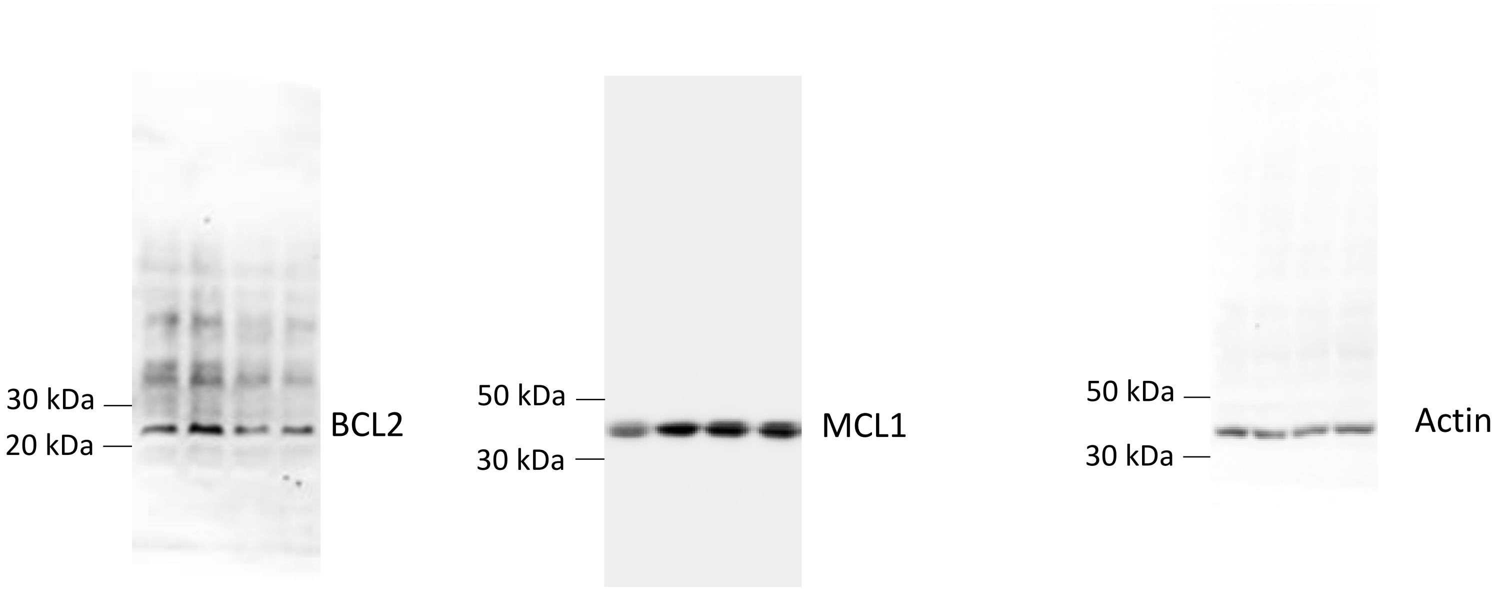

Figure S7

Whole images of immunoblots for Fig 6C

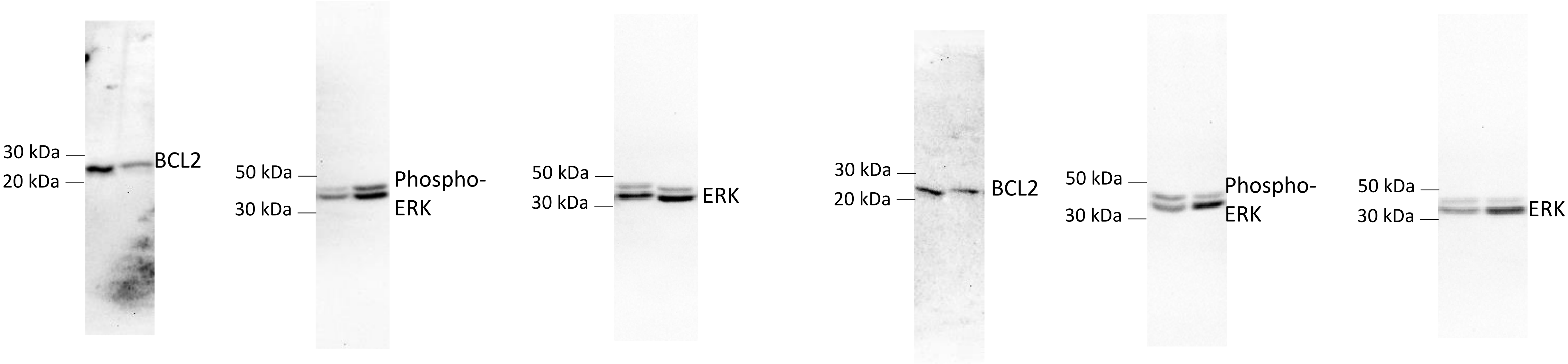

Figure S8

Whole images of immunoblots for Fig 6A

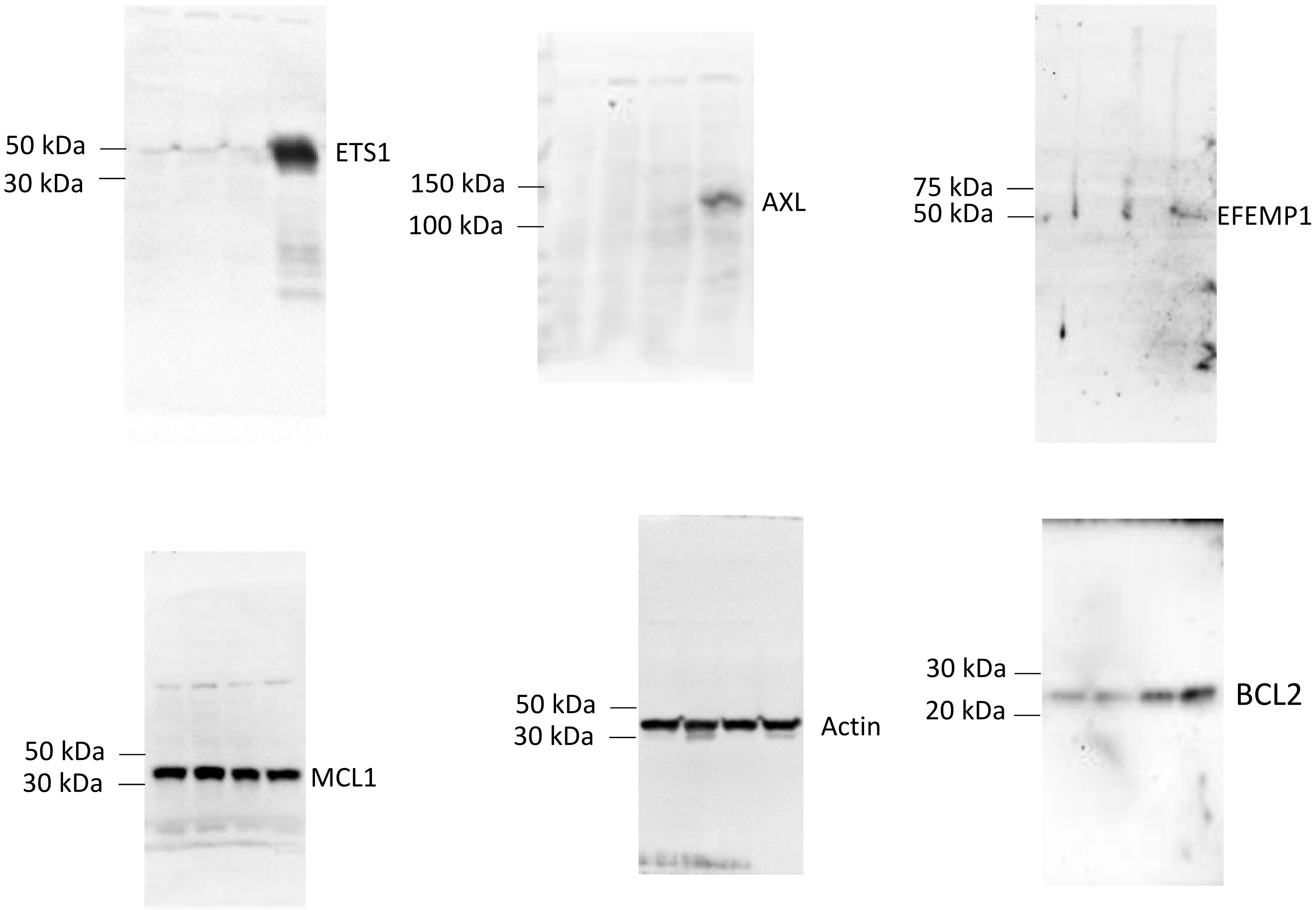

Figure S9

Whole images of immunoblots for Fig 6D

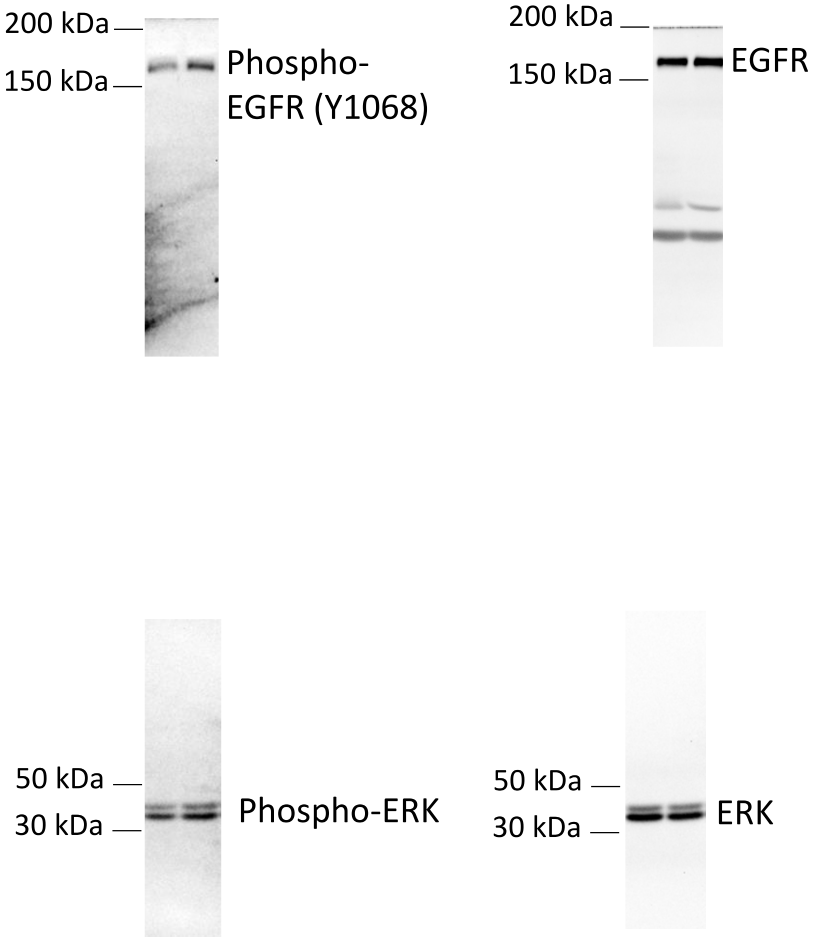

Whole images of immunoblots for Fig 6E

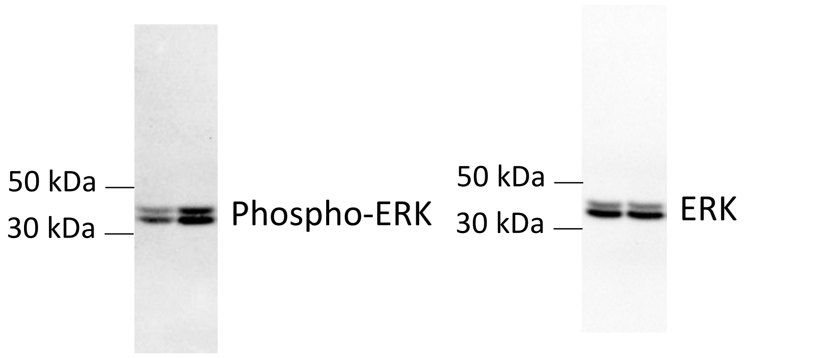

Figure S10

Whole images of immunoblots for Fig 6F

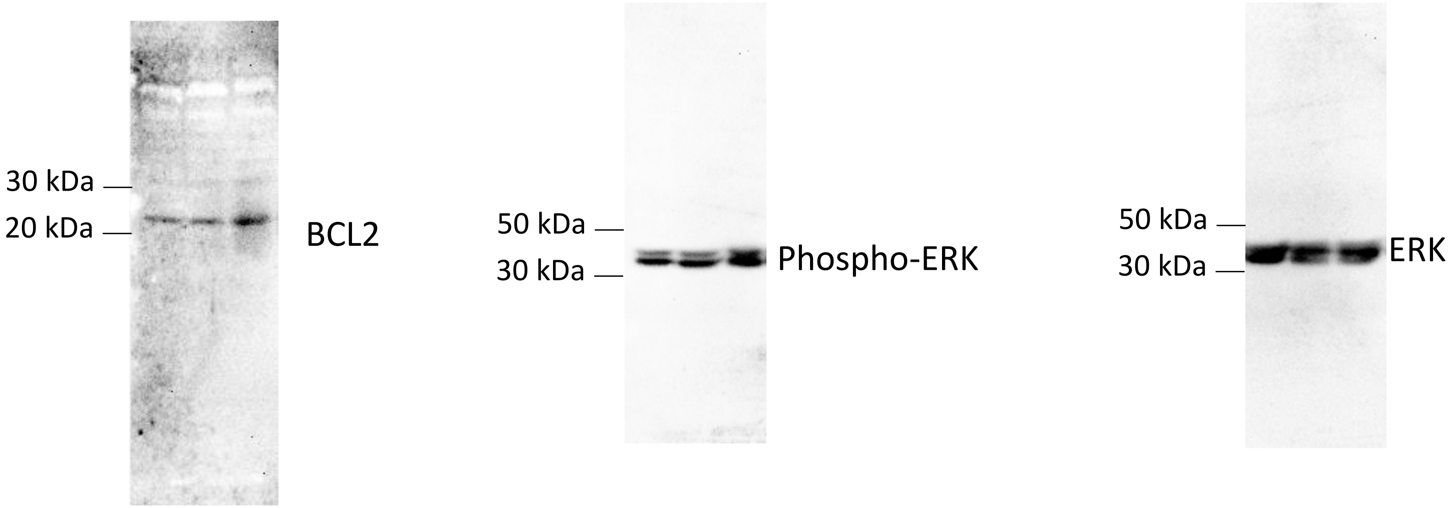

Supplement: Supplemental Data [file mmc1.pdf]
